# A Model for the Ultrastructure of Bone Based on Electron Microscopy of Ion-Milled Sections

**DOI:** 10.1371/journal.pone.0029258

**Published:** 2012-01-17

**Authors:** Elizabeth A. McNally, Henry P. Schwarcz, Gianluigi A. Botton, A. Larry Arsenault

**Affiliations:** 1 Department of Materials Science and Engineering, McMaster University, Hamilton, Ontario, Canada; 2 School of Geography and Earth Sciences, McMaster University, Hamilton, Ontario, Canada; 3 Canadian Centre for Electron Microscopy, McMaster University, Hamilton, Ontario, Canada; 4 Department of Pathology and Molecular Medicine, McMaster University, Hamilton, Ontario, Canada; Dalhousie University, Canada

## Abstract

The relationship between the mineral component of bone and associated collagen has been a matter of continued dispute. We use transmission electron microscopy (TEM) of cryogenically ion milled sections of fully-mineralized cortical bone to study the spatial and topological relationship between mineral and collagen. We observe that hydroxyapatite (HA) occurs largely as elongated plate-like structures which are external to and oriented parallel to the collagen fibrils. Dark field images suggest that the structures (“mineral structures”) are polycrystalline. They are approximately 5 nm thick, 70 nm wide and several hundred nm long. Using energy-dispersive X-ray analysis we show that approximately 70% of the HA occurs as mineral structures external to the fibrils. The remainder is found constrained to the gap zones. Comparative studies of other species suggest that this structural motif is ubiquitous in all vertebrates.

## Introduction

Bone is a composite material made up of two principal components: crystals of a mineral usually described as hydroxyapatite (HA) and fibrils constructed of co-aligned molecules of collagen. The spatial distribution and form of the crystals of HA has been a matter of some dispute since sections were first studied by electron microscopy in the 1950's [1]. An extensive literature points to much of the HA in bone residing in the 40 nm-long gap zones between the ends of collagen molecules, within the fibrils [2]–[9]. However the volume of the gap zones constitutes only 12 volume % of the fibrils. The mineral phase makes up ∼60 wt % of bone and therefore must constitute about ∼45 volume % of bone; therefore, about 73 total volume % of the mineral must reside outside the gap zones, taking up 33 volume % of bone. Earlier studies have suggested two possible solutions to this problem: a) that the HA crystals continue to grow beyond the ends of the gap zones and fill out part of the interior of the fibril [2], [3], [5], [10], [11], [12]; or b) some HA occurs between fibrils [9], [13]–[15]. Other recent studies appear to disregard this issue and assign all the mineral to the gap zone [16]–[18].

Other researchers have argued that the majority of the mineral in bone must be external to the collagen fibrils [19]–[24]. Lees, et al. [21] showed TEM images of cross-sections of mineralized turkey leg tendon in which much of the mineral forms a cladding around the fibrils. Referring to the mineral crystallites, they state: “If they are platelets viewed on edge they would be parallel to the fibril axis and surrounding the fibrils”. A predominance of extrafibrillar mineral was also inferred from models of the mechanical behavior of bone [22], from neutron diffraction studies of collagen in bone [20], and by analyzing bone using atomic force microscopy [25].

In an attempt to improve our understanding of the ultrastructure of bone, we have used a relatively untried method of sample preparation, cryo-ion milling, to prepare sections for TEM analysis. The sections were analyzed both by bright-field (BF) and dark-field (DF) methods as well as by scanning TEM (STEM) using high-angle annular dark field (HAADF) imaging. We used ion milling to prepare these samples because we had observed, both in images in the literature, and in our own preparations that conventional ultramicrotoming of fully mineralized cortical bone results in significant distortions of its internal structure. Ion milling and the related technique of focused ion beam (FIB) milling produce essentially no distortion because no stress is applied to the bone during milling. Cryo-ion milling has been previously used for TEM analysis of dentine [26]; Cressey and Cressey used an unspecified method of ion-beam thinning to visualize structures in modern and fossil bone [27], while Jantou et al. [28], [29] sectioned dentine (elephant tusk) for TEM using FIB. Nalla et al. [30] also used FIB to study human dentine but did not observe new ultrastructural features.

The present study initially focuses on the cortex of a single sample of human bone. We then show that analogous structures to those seen in human cortical bone can be observed in other human bones (including trabecular bone) and in bones of all other vertebrate species which we have studied.

## Materials and Methods

Our initial work was carried out on a section of the femoral diaphysis of a healthy 60 y old human male remaining from an allograft procedure. The procedures described for this sample were also used to analyze other samples to be described later. Fresh bone samples were preserved by treatment in formaldehyde solution (37% in water). Slices of bone about 1 mm thick were obtained using a slow speed water cooled diamond blade mounted in a South Bay Technology model 660 saw. These pieces were then dried in a graded series of ethanol baths (70, 80, 90, 96, 100% ethanol) for ten minutes three times at each concentration. The final drying step was an overnight soak in 100% ethanol.

The slices were thinned to ∼150 µm using SiC papers. Discs, 3 mm in diameter, were cut with a Gatan Model 601 ultrasonic disc cutter using 600 grit SiC powder. The centers of the discs were thinned to 30 µm using a Gatan Model 656 Dimple Grinder using 8 µm down to 0.75 µm diamond pastes. The samples were then milled on a Fischione 1010 ion mill equipped with a rotating cryostage, using a 3.0 kV, 3.0 mA beam of Ar^+^ ions oriented at 7° to the surface of the sample. During milling, the temperature of the sample was maintained at <−60°C using liquid N_2_. Milling was stopped when a small hole appeared in the center of the sample. The area immediately adjacent to the hole is ∼100 nm thick, thin enough to be electron transparent in TEM. A 5 nm coating of amorphous carbon was then applied using a Gatan Model 682 Precision Etching Coating System to reduce sample charging and heating in the electron microscope.

TEM images were obtained using a Philips CM-12 microscope operated at 120 kV. Images were recorded using both Kodak SO-163 electron sensitive film and a Gatan Orius CCD. Film negatives were scanned into digital formats for analysis. Scanning TEM (STEM) images and energy dispersive X-ray spectrometric (EDXS) data were obtained using an FEI Titan 80–300 Cryo TEM operating in HAADF mode at 300 kV. The probe convergence angle was 8 mrad and the HAADF detector inner collection angle was 35 mrad, operated at a camera length of 152 mm. EDXS counts were corrected for matrix effects using ZAF software (Oxford Instruments).

Most images reported here are bright field (BF), that is formed by the electrons transmitted through the section; part of the electron contrast observed is due to diffraction scattering by crystals in the sample. In addition, we recorded some dark field (DF) images which are formed by electrons which have been Bragg-scattered from a specific lattice plane of HA.

While the samples were being viewed in TEM, care was taken to observe if any damage was produced by the incident electron beam. This occurred only after long exposure times (>20 min) and could be easily recognized by changes in the appearance of collagen-rich areas. Initially, however, samples which had been ion milled showed no such evidence of damage.

Because the samples used in this study had been treated with formalin, there might be some concern that structural changes to the bone may have resulted. Some samples of bovine bone that will be discussed later were treated both with formalin and by the more conventional method of treatment in ethanol. No differences in the appearance in TEM images of the sample were observed between the two treatment methods. We also measured the dimensions of rectangular blocks of cortical bone before and after prolonged immersion in formalin (up to one month). No detectable changes in the dimensions of these blocks were observed. We also note that the images which we obtained are very similar to those obtained on samples of elephant dentine (ivory) by Jantou-Morris et al. [28], [29], suggesting that the methods of preservation used here have not induced significant structural changes. An additional test (measurement of the D spacing of the banding in collagen) described below confirms that even at the nanometer scale there has been no deformation of the collagen. In any case, the focus of this paper is on the structure of the mineral phase which would presumably not be affected by any chemicals used to preserve the bone.

Sections were cut parallel and perpendicular to the long axis of the femur. In this way we expected to obtain ion-milled sections in which, respectively, collagen fibrils would be either lying horizontally in the plane of the section, or were cut approximately normal to the fibril axes. This expectation is based on previous microscopic studies of bone showing that osteons are slightly inclined parallel to the long axis of the bone [31] and that fibrils are arranged at small inclinations to the axes of osteons [32]. Obviously, in any given ∼1 µm^2^ area viewed in TEM, the orientation of fibrils might deviate significantly from these expected orientations.

The slices of bone used to prepare perpendicular TEM sections were of the entire thickness of the cortex, and discs cut out for analysis were taken at random locations across the this section but did not include the periosteal or endosteal surfaces. Likewise, the longitudinal sections were cut at randomly located depths in the interior of the cortex. There did not appear to be differences in the nanoscale structures seen in TEM images depending on position within the cortex.

For comparative purposes, some sections of bovine bone were sectioned by ultramicrotoming in the electron microscopy facility of the Faculty of Health Sciences, McMaster University. A diaphyseal section of a bovine femur was obtained from a local butcher, cleaned of all muscle tissue and marrow, defatted in a mixture of chloroform and methanol (1∶1) and then stored in formalin solution for 24 h. Pieces of cortical bone 2–3 mm in maximum dimension were cut oriented either parallel or perpendicular to the femoral axis. These were dehydrated in alcohol as described above. The samples were embedded in Spurr's resin, then sectioned into slices between 70 and 90 nm thick using a Leica UCT ultramicrotome and a diamond blade angle of 45°. The slices were floated in water onto Formvar coated copper grids. The same procedures was also used to prepare a section of the femur of a 6 month-old mouse (*Mus musculus*) provided by Dr. M. Glogauer, University of Toronto.

The samples of human femoral bone from an allograft specimen were used by permission of the Research Ethics Board of the Faculty of Health Sciences, McMaster University. Samples of human bone donated by Dr. M. Ghert, were obtained with the written consent of the patients and their use was carried out with the specific permission of the Research Ethics Board of the Faculty of Health Sciences. Use of samples of mouse bone donated by Dr. M. Glogauer was approved by University of Toronto Animal Ethics Committee who assigned an animal protocol number of 20008891.

## Results

The following are observations made on sections of the human femoral diaphysis.

### Longitudinal sections

**Figure 1:**
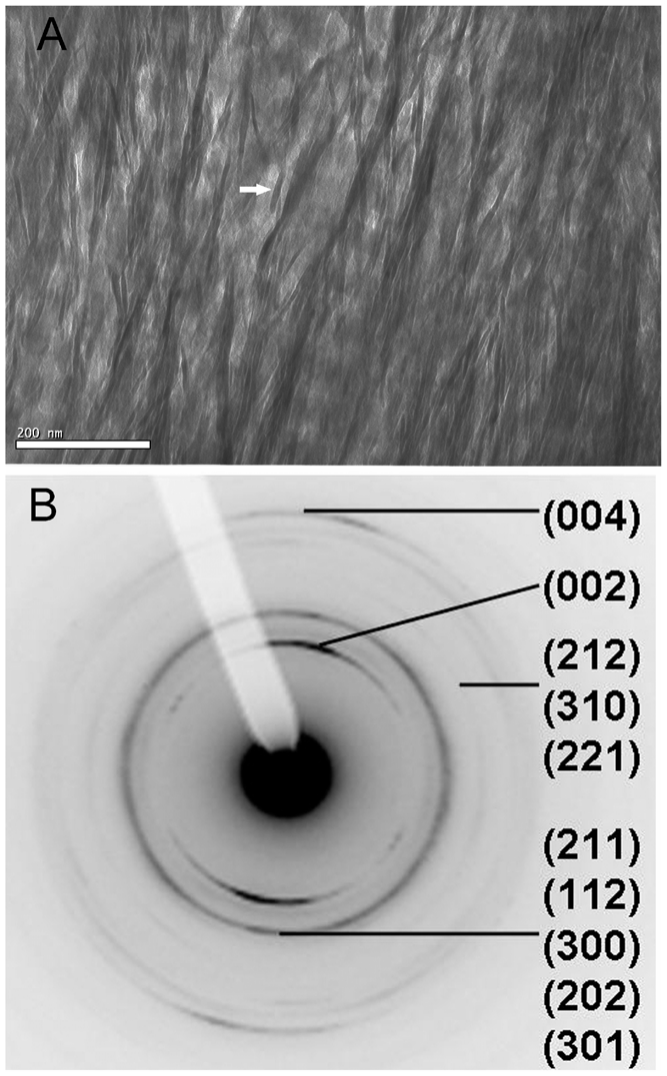
Human femur sectioned parallel to long axis of femur. a) bright field (BF) image: faint bands oriented NW-SE, repeated every 68 nm, denote concentration of HA in gap zones in collagen fibrils which run perpendicular to the bands. Perpendicular to the bands are ∼23 nm wide bundles spaced ∼50 nm apart comprised of clusters of linear features (arrow) 5±1 nm wide, and up to 200 nm in length; b) selected area diffraction pattern indexed to HA; note that 00l reflections form arcs indicating preferred orientation of the c axes of the HA parallel to the to the fibrils, while other reflections form complete circles, lack of alignment of the a and b axes.

**Figure 2:**
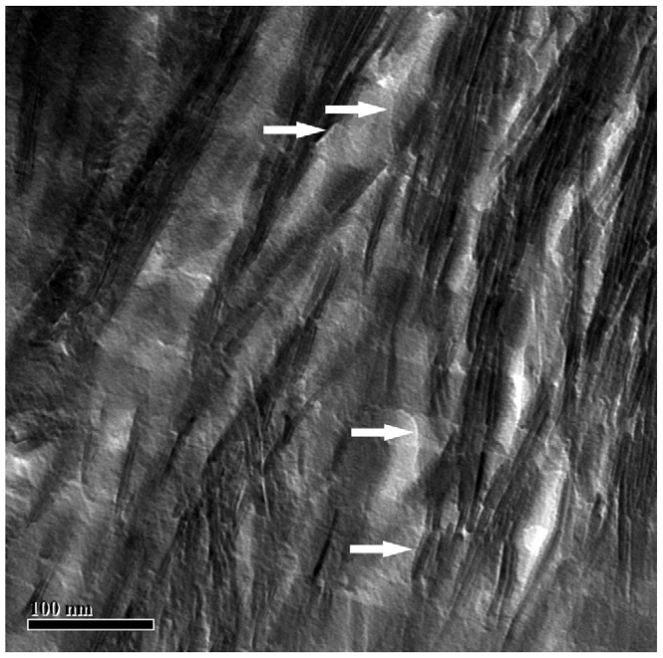
Sharply defined borders between gap zone and overlap zone. Bright field TEM image of longitudinal section of human bone. Image shows sharp border between high-contrast gap zones and low-contrast overlap zones. There is no evidence that the higher-contrast material (presumably HA) penetrates into the overlap zone. Arrows point to possible boundaries of constituent crystals (see *Conclusions*).

Bright field (BF) images were obtained of samples cut parallel to the long axis of the femur (referred to here as longitudinal sections). As expected, these displayed the periodic banding characteristic of Type I collagen fibrils (Figure 1), as shown in other studies (for example [29], [32], [33]). The contrast of the bands is generally assumed to be due to the presence of crystals of HA within the gap zones of the fibrils. The axes of the fibrils must therefore be oriented perpendicular to the banding. Between the gap zones are lighter (low contrast) zones representing the overlap zones where HA is presumably less abundant in the fibrils. In some images, we see that the boundaries between gap and overlap zones are relatively diffuse, whereas in a few images these boundaries are sharply defined (Figure 2). The spacing of the gap zones (D-period) was measured in nine randomly selected longitudinal sections, and gave an average of 68.0±4.3 nm. This is comparable to values observed in other studies [10], suggesting that use of formalin as a preservative has not altered the dimensions of the collagen structure. The banding is in registry between adjacent fibrils, forming continuous stripes that extend over several hundred nanometres as observed in mosaics compiled from multiple images (not shown).

In Figure 1 we also observe dark elongated features running perpendicular to the banding, and therefore parallel to the collagen fibrils. The dark features are spaced about 50 nm apart and extend along their long axes well beyond a single repeat of the banding, to distances of several hundred nm. Similar features are present but much less well defined in earlier images of sections prepared by ultramicrotome (e.g. Figure 3a of [1]). Essentially identical features are seen FIB sections studied by Jantou et al. (Figure 4a of [28]) where they are referred to as “apatite crystals”.

**Figure 3:**
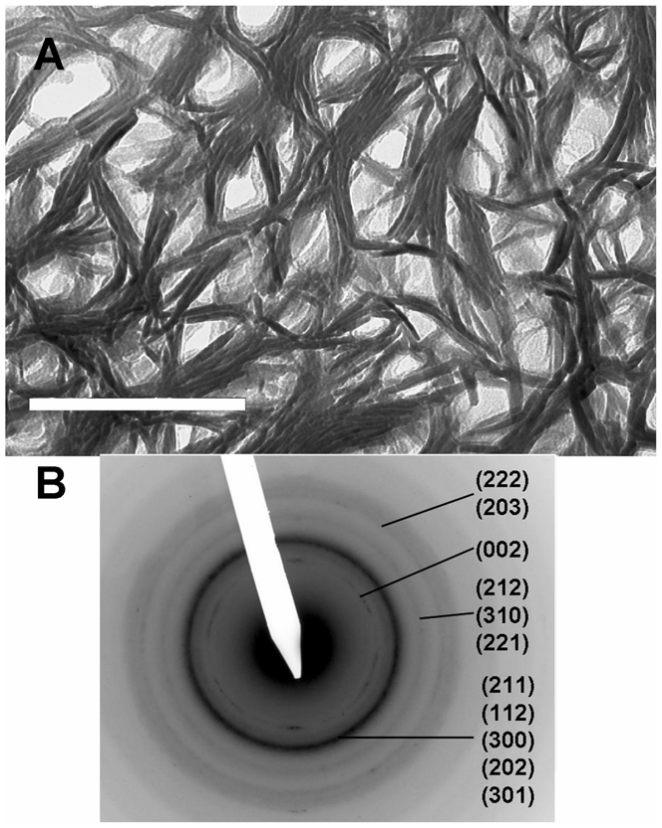
TEM image of cross-section of femur. a) Bright-field image; scale bar = 100 nm. Low-contrast (light) areas surrounded by dark linear features are believed to be sections through collagen fibrils, many of which have been punctured by ion beam during ion milling; b) selected area diffraction pattern; note spotty 00l rings confirming that c-axes of HA are oriented normal to plane of section.

**Figure 4:**
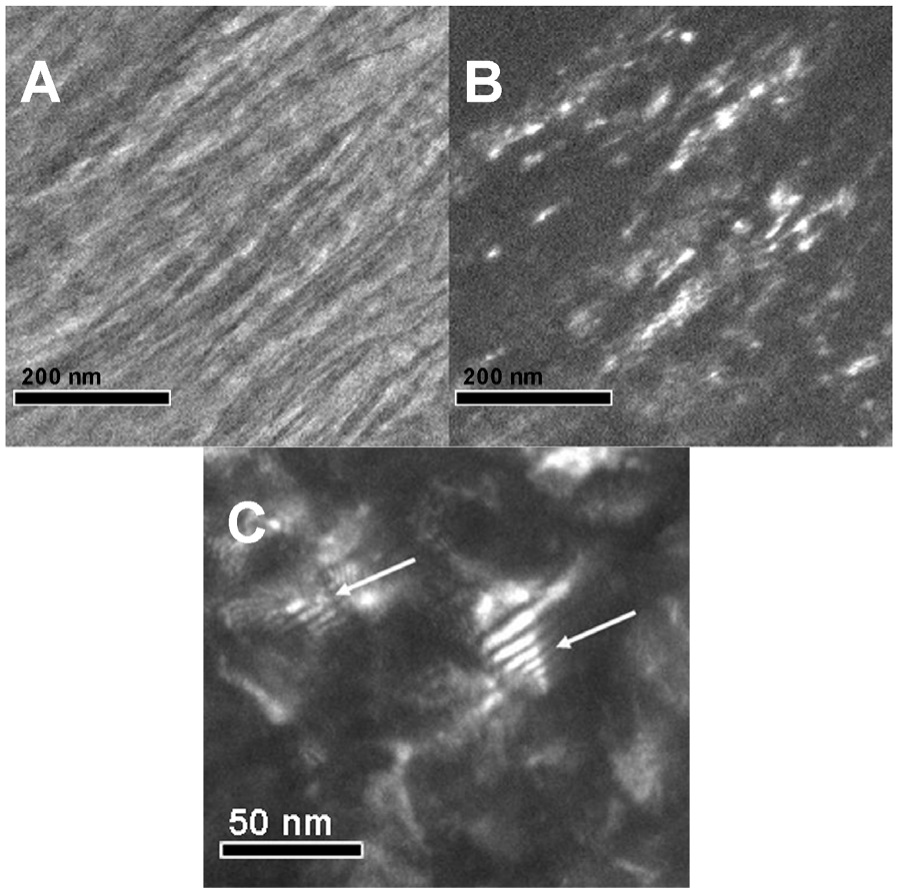
Dark field images. (a) bright-field and (b) dark-field images (using 002 reflections) of same region in a sample of human bone cut parallel to the long axis of the bone. Bragg reflections from the 002 planes of HA are concentrated along the long dark structures, showing that they contain crystals of HA. Note lack of 002 reflections from gap zones. c) Dark field image of a second area showing Moiré fringes in area between lanes of mineral structures that are not visible in this image.

Closer inspection of these dark features shows that they are in fact bundles of smaller linear dark structures (Figure 1) which measure 5±2 nm in width. Individual structures are internally featureless. They are up to 200 nm in length but in general it is not possible to resolve the terminations of a given structure and thereby determine its apparent length. Individual terminations of these structures, where they can be seen, appear to be blunt or almost squared off.

Between the bundles or lanes of dark structures, the gap and overlap zones are more clearly discernible. We also note that the overlap zones show a significant electron contrast, not much less than that of the gap zones. Consequently, all the longitudinal images have a dark cast, as if an electron-dense component was widespread over the entire section. If the overlap zones contained only collagen, we would expect a much lower degree of contrast, due to the much lower average atomic number of collagen compared to HA.

### Perpendicular (cross) sections

TEM images of sections cut so that the plane of the section is approximately perpendicular to the axis of the femur (referred to here as cross-sections) are strikingly different from longitudinal images (Figure 3). In all sections cut in this orientation we see an open, lacy pattern consisting of circular to elliptical, low-contrast “holes” some tens of nm in diameter, which are tangentially surrounded by dark (high electron contrast) features. As in the longitudinal sections, the dark features are seen to be composed of individual dark, internally featureless structures ∼5 nm thick.

The holes are partly filled with material of much lower contrast than the surrounding structures. In some holes, this material extends across the entire width of the hole, whereas in others the hole is partly or entirely vacant empty except for scraps of this lower contrast material which remain around its edges or in one corner. Comparison of Figure 1 and Figure 3 shows that the electron contrast in the non-vacant holes is substantially less than in the overlap (lowest contrast) zones of the longitudinal sections.

Similar images were presented by Jantou et al. [28], [29] for TEM images of focused ion beam sections of ivory dentine. They also refer to the dark structures around the margin as “apatite crystals”. Cressey and Cressey also showed such structures using ion-milled sections of bone [27]. Earlier, Prostak and Lees, studying mineralized turkey tendon [19] had shown similar, but less well-resolved images of extrafibrillar “crystallites” surrounding collagen fibrils.

### Electron diffraction studies

Selected area diffraction (SAD) analyses have been widely used to characterize the nature of the mineral phase and the orientation of the crystal lattice [16], [34], [35], [36]. SAD analyses of both longitudinal and cross-sections display the characteristic diffraction pattern of hydroxyapatite (Figures 1b and 3b).

In SAD patterns taken from the longitudinal sections the 002 and 004 reflections consist of two arcs which subtend angles of 36° and which are centered around the direction of the collagen fibrils. Incomplete 00l rings (arcs) have been previously observed in SAD patterns obtained from bone [35], [37]. The angular dispersion of the arcs is similar in magnitude to the angular dispersion (herringbone pattern) of the dark structures in the BF images (Figure 1), suggesting that the crystals responsible for 00l arcs may be in these structures. It is also possible that angular dispersion of HA crystals inside the gap zones could be responsible for this feature. Other reflections do not show a preferred orientation.

SAD patterns from cross-sections show almost no 00l reflections (Figure 3b), suggesting that these lattice planes are primarily oriented normal to the plane of the section. This is consistent with the earlier observation that the 002, 004 reflections are concentrated into narrow arcs the midpoints of which are aligned with the fibril axes. The other hkl reflections are uniformly distributed and form continuous circles.

### Dark field images

Dark field (DF) TEM images are obtained using electrons which have been Bragg-scattered from a specific lattice plane in a crystal. Dark field imaging allows us to localize the crystals which were responsible for forming the corresponding reflection in the SAD pattern and thus to learn about the distribution of the crystals of HA in the sample. Figure 4 shows a typical pair of bright field (BF) and dark-field (DF) images from a longitudinal section, using the 002 reflection of HA. The image shows that the 002 reflections are entirely concentrated along the elongated thin dark structures which we previously identified. We conclude therefore that these ∼5 nm thick structures are composed of crystals of hydroxyapatite and that most of the HA is in those structures.

The entire length of a mineral structure is never visble in DF (Figure 4). Instead, discrete segments appear bright while the remainder is dark. If the mineral structures were single crystals then we would expect them to be visble along their entire length unless they were bent so that the lattice plane being used to generate the DF image was locally misaligned. To test this, we could observe the section in DF illumination while rotating the objective aperture of the electron microscope along a single hkl diffraction ring, which would allow us to detect crystals in different orientations in the sample. Specifically, if the mineral structures were single crystals then we would expect that the zone of illumination would move continuously along the mineral structure. For this purpose we initially used the 211 ring and did observe the visble portion of each mineral structure move continuously along the structure. We note however that this ring is the unresolved combination of the 211, 112 and 300 Bragg reflections, each of which is of comparable relative intensity (100∶60∶60 respectively). Therefore the successive visble areas could represent different crystals aligned so that one of these three reflections was in scattering orientation. Therefore, we used instead the 002 reflection which, as noted above, is not a complete ring but an arc of 36°. As the aperture moved along the 002 arc, successive portions of the same mineral structure were alternately visble, discontinuously. This supports the notion that the mineral structures are polycrystalline rather than bent single crystals.

The gap zones are not visble in DF images constructed using the 002 arc. However, DF images of longitudinal sections using the 211+112+300 ring (“211+”) shows faint but well-resolved reflections within the gap zone indicating the presence of HA crystals.

In some DF images using either the 002 or 211+ reflection, Moiré fringes occur in patches a few tens of nm across, scattered through the image (Figure 4c). These appear to occur in areas between the elongated dark structures.

### Energy-dispersive X-ray spectroscopy (EDXS)

**Figure 5:**
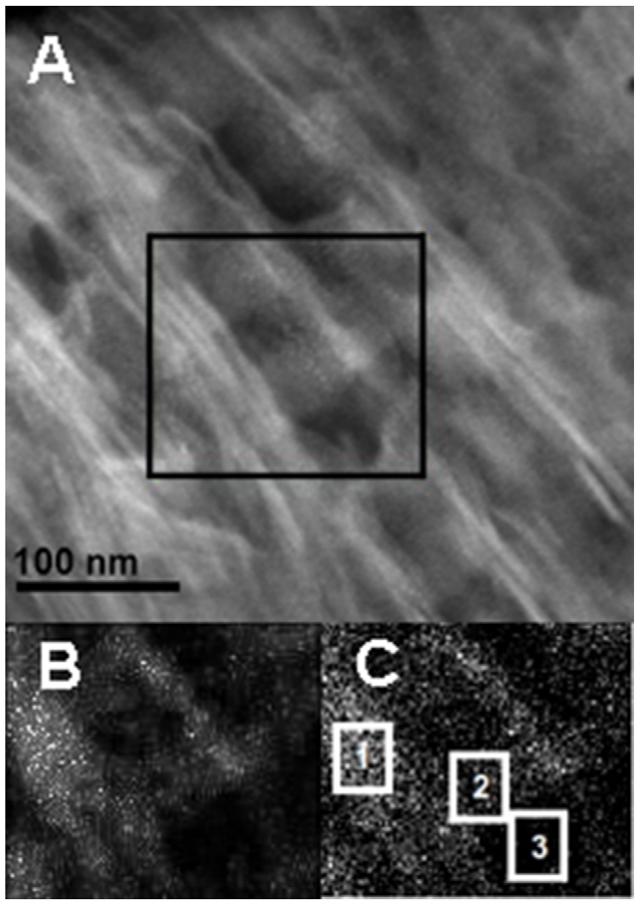
STEM annular dark field image and matching EDXS maps of longitudinal sections of human bone. a) STEM image; Box outlines area shown in EDXS map, b) EDXS map of Ca distribution of area bounded by box in A. Lowest levels of Ca are over overlap zones, highest levels are over the NW-SE trending mineral structures. c) Boxes 1, 2, and 3 are areas of analysis described later in the text.

EDXS measurements allow us independently to determine the spatial distribution and abundance ratios of calcium (Ca) and phosphorus (P). These measurements were carried out in scanning transmission electron microscopy (STEM) mode on an FEI Titan 80–300 microscope. Figure 5 shows a typical element map obtained from a longitudinal section along with the corresponding STEM image. The density of points in each image is proportional to the concentration of the element. Calcium (shown) and phosphorus (not shown) levels are elevated over the elongated dark structures as well as over the gap zones and lower over the overlap zones. The Ca∶P atomic ratio in both regions ranges from 1.46∶1 to 1.75∶1, consistent with the expected ratio of 1.67∶1 for HA. Count rates are higher over the dark structures than over the gap zones, as will be discussed later.

The elongated dark structures seen in both longitudinal and cross-section are apparently largely composed of the mineral component of bone (hydroxyapatite). We have not, at this point in the discussion, defined what is their full three-dimensional form but, to simplify further discussion of them, we shall refer to them as *mineral structures*, a term which leaves open the issue as to whether they are needle-like or plate-like or of some other form.

### Dimensions of mineral structures

Cortical bone is constantly being remodeled by replacement of older bone by Haversian systems (osteons). In the course of studying the femoral diaphysis investigated here. We examined approximately thirty different sections, in each of which two or more osteons were present; therefore parts of multiple osteons were analyzed, even in the same ion milled section. Inevitably no two osteons would have been caught at the same stage of their life-history. Nevertheless we observed a surprising degree of uniformity in the appearance and dimensions of the mineral structures.

In tables 1 and 2 we give the ranges for these dimensions based on detailed measurements of representative ion-milled sections. Note that the precision of these measurements is limited by the pixel size in the images, which depends on the magnification of the image. Typically pixel sizes were between 0.18 and 0.34 nm.

**Table 1:** Measurements of Mineral Sections in Longitudinal Section Image (Figure 1).

Measurement Type	Value	n
Thickness	4.8±0.9 nm,	25
Distance between bundles (lanes) of mineral structures	53±13 nm	7
Widths of bundles of mineral structures	23±7 nm	5
Lengths of mineral structures	Shortest ≥40 nm Longest >200*	

*Note – ends of mineral structures are obstructed by other structures or extend out of the focal plane of the image.

**Table 2:** Measurements of Mineral Sections in Cross-section Image (Figure 3).

Measurement Type	Value	n
Thickness of mineral structures	4.9±0.9 nm	25
Lengths of mineral structures	68±18 nm	11
Average closest distance between two holes, viewed in section	27. 7±10.4	11
Average hole major axis	54.6±23.9 nm	15
Average hole minor axis	36.3±18.1 nm	15
Average hole eccentricity (major axis/minor axis)	1.6±0.4	15
Average of major and minor axes	45.4±22.8 nm	15

The measured range of dimensions for the cross-sectioned samples (table 2) is compatible with a population of fibrils which are actually cylindrical in cross-section when cut normal to their axes. The apparent elliptical form would be a consequence of cutting sections at a small angle to the fibril axis. However the dimensions are also compatible with sections cut through fibrils with truly elliptical cross-sections.

### Comparison with images of other samples

The images of human femoral cortical bone discussed so far are similar to published images of obtained by TEM imaging of focused ion beam (FIB) samples of elephant dentine [28], [29].

**Figure 6:**
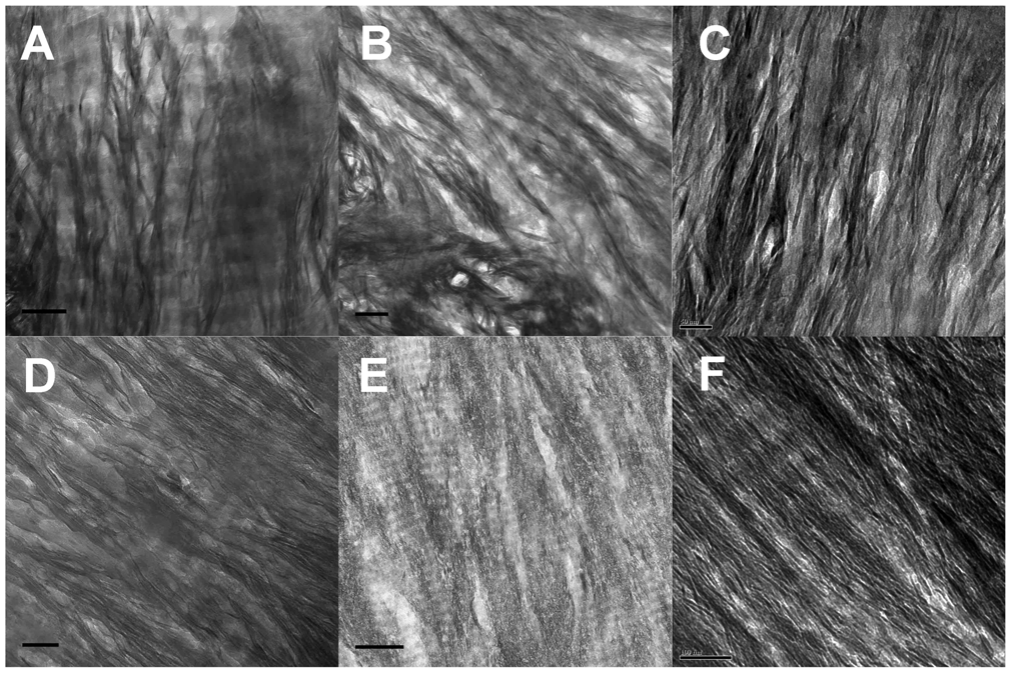
Longitudinal TEM bright field images of other bone samples. a) femoral cortex of 19 y-old healthy male (allograft specimen) Scale = 100 nm; b) allograft remainder of 60 y-old male; scale = 100 nm; c) bovine femur; scale = 50 nm; d) elephant (mammoth [*Mammuthus sp.*]), c. 10,000 y old, Siberia; scale = 100 nm; e) Salmon (*Oncorhynchus* sp.)vertebra; scale = 300 nm; f) femur of a 6-month-old mouse (*Mus musculus*); scale = 100 nm.

In addition we have used ion milling to prepare longitudinal sections from other bone samples: two human samples, cow (bovine), elephant (10,000 y-old mammoth), mouse, and vertebra of a fish. Figure 6 shows bright field TEM images obtained from these samples. All the samples except the elephant were prepared according to the procedures described earlier in this paper. The elephant bone had apparently been frozen since death; a section was cut without additional preparation of the material. The murine femur, too small to be sectioned by ion milling, was sectioned by ultramicrotome. The fish vertebra was also prepared in this manner. Additional comparative images of bovine bone are shown below accompanying the discussion on ultramicrotoming.

For each of these samples, TEM analysis of ion milled sections displays structures qualitatively similar to those seen in the human femur. Furthermore, the dimensions of the mineral structures and their bundles are similar to those in the human femur. We infer that features observed in the sample of cortical human bone are generally present in all mammalian bone and may also be widespread in other vertebrates.

## Discussion

Using cryogenic ion milling, we prepared sections of randomly selected 3 mm discs cut from the cortex of the same piece of bone (human femur), and used these to obtain TEM images taken approximately parallel and perpendicular to the axes of the collagen fibrils. The orientation of the sections is indicated by the presence of well-defined gap zones and overlap zones in the longitudinal (parallel to fibril) sections, and the absence of such structures in the cross-sections. Thus we should think of these as being images of the same overall structure viewed from two aspects approximately at right angles to one another.

**Figure 7:**
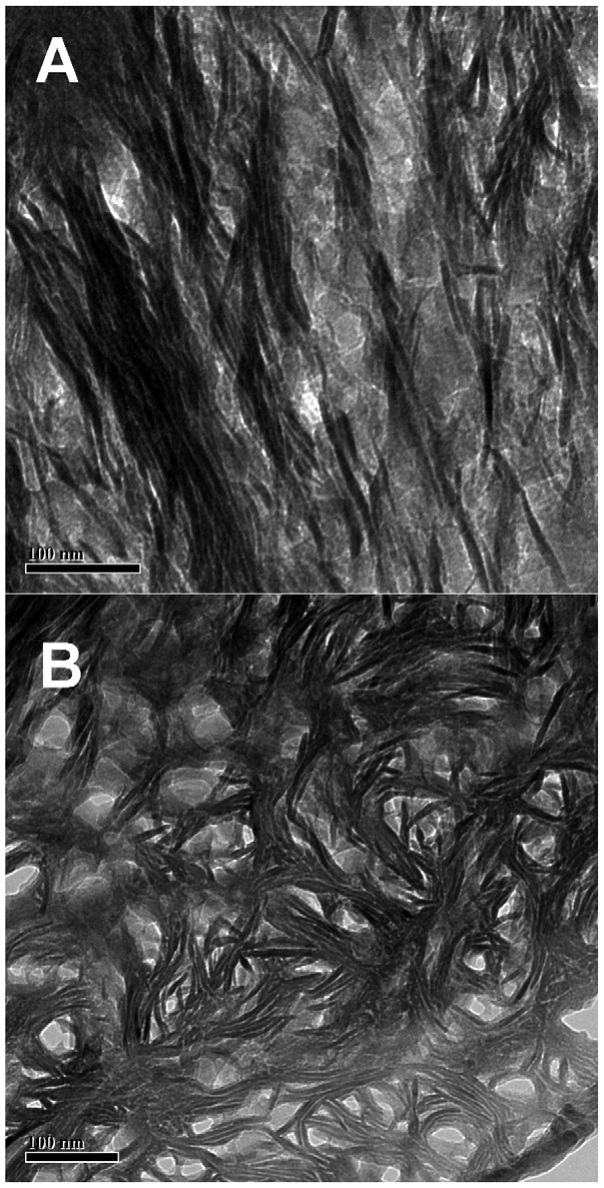
Series of bright-field TEM images of a single section at varying orientation (tilt). The section was initially cut at 45° to the axis of the femur, and tilted while being viewed in the electron microscope: a) tilted to −45°, showing bands of mineral structures aligned parallel to fibrils; b) tilted to +45°, showing open, lacy structure of mineral structures.

Figure 7 shows a series of TEM images made on a section cut at 45° to the axis of the femur; the images were obtained by tilting this section from −45° to +45° so that the same section was being viewed either parallel or perpendicular to axis of the bone. Figure 7a (−45° tilt) shows bundles of mineral structures similar to those obtained in our longitudinal sections, while Figure 7b (+45° tilt) resembles our cross-section views, with a lacy pattern of mineral structures surrounding holes which we presume to be sections through collagen fibrils. Clearly we are seeing the same structures in each view, from different aspects.

We infer from these and our other studies that the mineral structures visible in the longitudinal sections must be identical to the HA-rich structures seen to be wrapping around holes in the cross-sections. We can then ask: what is the full three-dimensional form of these mineral structures? A possible form for the complete mineral structures that could generate these two kinds of images would be long plates (laths) ∼5 nm thick and ∼70 nm wide and with a third dimension ranging to >200 nm. These plates must be oriented with their long axes parallel to, or at small angles to the fibrils. The dark-field images suggest that the mineral structures are not single crystals of hydroxyapatite but rather they are polycrystalline, the individual crystals being on the order of a few tens of nm long. The spacing between adjacent mineral structures is at most 0.1 nm; the gap between them can be barely resolved in some images, where the mineral structures are fortuitously oriented to permit this view.

Some HA also occurs in the gap zones but its form is much less well-resolved than the mineral structures. No discrete crystals can be recognized although in DF images using the 211+ reflection we see spots corresponding to individual crystals in the gap zones. The presence of HA in the gap zone is also attested to by EDXS maps showing a higher concentration of Ca and P than in the adjacent overlap zones, and by the enhanced electron contrast of the gap zones seen in BF images. The boundaries between gap- and overlap-zones are usually blurred, but in a few images (e.g. Figure 2) they are sharp and well-defined. This variation in appearance of the overlap/gap-zone contact is expected if the contact is in fact well-defined but viewed at an inclined angle to the axis of the fibril. Such inclined orientations are expected to occur, since the sections are not cut precisely parallel to the fibril axes.

We also propose that the holes surrounded by the mineral structures, as seen in the cross-sectional views, are collagen fibrils viewed in cross-section. As noted above, some of the holes are wholly or partly vacant, whereas others are filled with low-contrast material that could be collagen. The partially or wholly vacant holes may be a result of the much more rapid erosion of collagen compared with the adjacent mineral structures during ion milling. Inasmuch as the fibrils must be visible somewhere in these cross-sectional views it seems likely that these low-contrast holes are the loci of the fibrils. Their low electron contrast confirms that they contain much less mineral than the surrounding structures. Interestingly, virtually none of the holes are vacant in the cross-section-like view of the 45° section (Figure 7b); contrast this image with Figure 3a. Presumably the inclined aspect of the fibrils to the Ar^+^ ions in the ion mill in this case partially protected them from complete erosion of the collagen.

**Figure 8:**
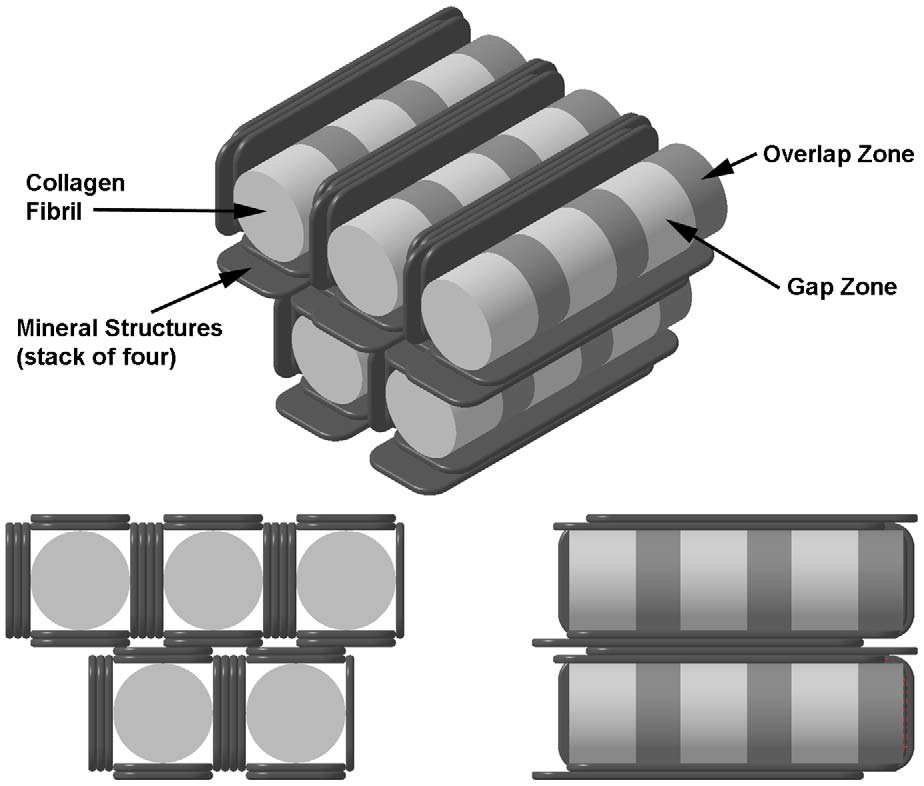
Simplified model of bone mineral and collagen fibrils in fully dense cortical bone. Model is based on measurements from longitudinal and cross-sections of cortical bone. 45 nm-diameter collagen fibrils are shown with 40 nm-long gap zones (light) and 27 nm overlap zones (dark). Plate-like mineral structures 5 nm thick, 65 nm wide, 200 nm long tangentially surround the collagen fibrils. Mineral structures are shown stacked 4-deep between adjacent fibrils, as inferred from average inter-fibril separation and 5 nm thickness of mineral structures. A more accurate model would show mineral structures more completely surrounding each fibril (as seen in Fig. 3a).

A possible model for the ultrastructure of bone is sketched in Figure 8. Here we have shown only plates (mineral structures) oriented in two directions around the fibrils, whereas in the complete structure the mineral structures form a complete carapace encasing the fibrils. The model illustrates that on average there are about four mineral structures separating each adjacent pair of fibrils; this estimate is based on measurements of the average distance between fibrils (27. 7±10.4 nm). Figure 8 also fails to represent the tendency of the long axes of the mineral structures to be oriented at small angles to the fibril axes (up to 36°, based on the angular spread of the 002 reflection in SAD images).

This model for the structure of bone can also be considered in relation to specific features of the TEM images. In the longitudinal images (e.g., Figure 1) we noted that the mineral structures tended to occur in clusters or lanes spaced about 53 nm across. In our model bundles of mineral structures are located between adjacent fibrils; the average distance between these bundles should be equal to the average width of the fibrils which we found to be 45±23 nm. This agrees closely with the spacing of the lanes, as expected.

Also, the model would lead us to expect that during TEM imaging of longitudinal sections, the electron beam would pass through one or more stacks of mineral structures oriented parallel to the plane of the section (the ion-milled sections are ∼100 nm thick). This would account for the dark cast seen in images of the inter-lane regions, much higher contrast that we see, for example in the holes in cross-sectional views. The dark cast extends over the overlap zone; also, EDXS images of the overlap zone show significant concentrations of Ca (Figure 5) and P. While in principle this could be a result of the presence of HA extending into the overlap zone, the crisp boundaries between overlap and gap zones seen in some BF images (Figure 2) argues against any extension of HA crystals from one zone into the other (contrary to the suggestion of Landis et al. in Figure 6 of [3]).

### Estimate of amount of extrafibrillar mineral

Overall, the model we have envisioned posits that large amounts of mineral are located in well-defined structures outside the fibrils. The question remains what fraction of the total HA in bone lies outside the fibrils. In an attempt to answer this question we will use EDXS data obtained from analysis of a longitudinal section (Figure 5). This is preferable to use of similar data from a cross-section for two reasons: a) the thickness of the cross-section is unknown and the EDXS signal intensity over the holes (i.e., spaces surrounded by mineral structures) will depend on the elevation of gap zones within a given fibril with respect to the plane of the section; and b) some of the holes in our sections are partly vacant due to loss of fibrillar material during sample preparation, suggesting that all of them are at least slightly eroded by the Ar^+^ beam.

**Figure 9:**
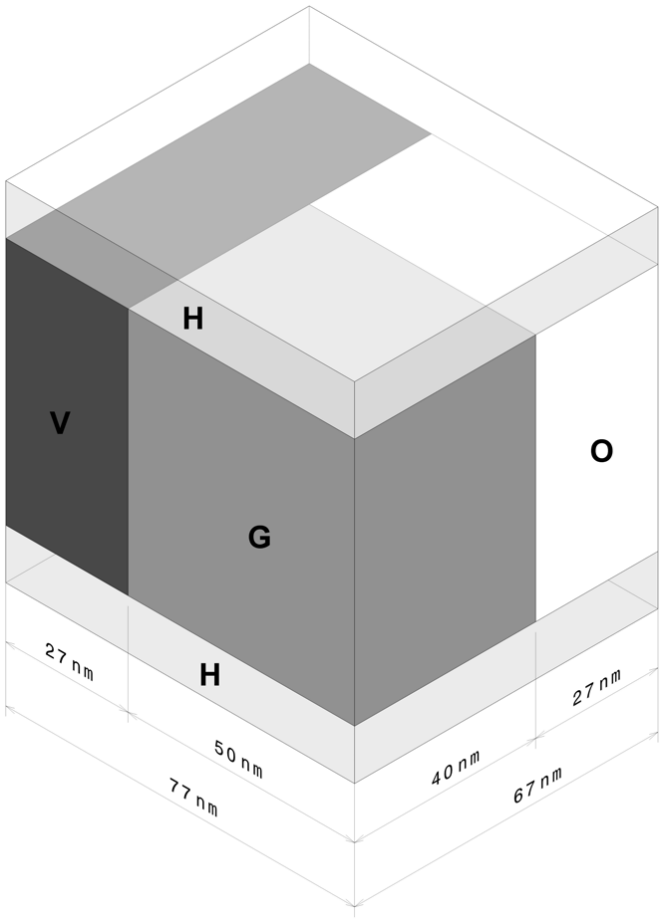
Simplified isometric model of unit volume of bone. The model shows the composite fibril/mineral structural makeup of cortical bone for the purpose of using EDXS data to estimate spatial distribution of mineral. The subvolumes H, G, V and O are identified in the text. EDXS X-rays are recorded emerging from top of this structure.

To interpret the EDXS data we will make use of a modified version of the simplified model for bone structure. Figure 9 shows a unit volume of total bone subdivided into compartments labeled as follows:

H: over- and under-lying mineral structures, oriented parallel to the plane of the section.

V: mineral structures oriented normal to plane of section (vertical).

G: gap zone, containing mineral.

O: overlap zone, containing only collagen; as explained above, we see no evidence that mineral from the gap zone extends into the overlap zone, nor should it according to the gap-zone model [6].

Note that we neglect the structures which lie at shallower angles to the plane of the section and complete the wrapping of the fibril by mineral structures; their Ca counts would be included in either V or H.

The EDXS count-rates record X-rays emerging from the upper surface of this model volume. We obtained data for Ca count rates for selected 100 nm^2^ areas of three representative longitudinal sections. A typical example is shown in Figure 5b. Ca counts were recorded emerging from the following selected areas:

1 = mineral structure (V)

2 = gap zone (G)

3 = overlap zone (O)

These identifications were confirmed by inspection of the corresponding BF images for these areas as shown in Figure 5a. For the first two of these regions we can see by inspection of Figure 9 that the observed counts must also include Ca counts originating in the over/underlying mineral structures (volume H), whereas *all* of the Ca counts in area 3 come from H, since the overlap zone of the fibril is assumed to contain no HA.

We will also assume that the counts/nm^2^ over a given area are proportional to the total amount of HA below the area, because the depth of stimulation of X-rays from the sample encompasses the entire thickness of the sample. This is because the depth required for 95% extinction of Ca Kα X-rays is approximately 1.7 µm [38], many times thicker than the section (∼100 nm).

We can then use these measurements to calculate the relative amount of HA in each region of the composite bone structure and specifically to determine X_ext_, the fraction of mineral that is external to the fibril.

Let C(*i*) = count rate (counts/nm^2^) from a given area i = [observed counts in area]/100. The respective areas of interest are G,V and O;

Then we can correct the counts for G and V for the contribution by H (over/underlying MS) as follows:
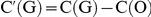


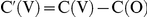
The amount of Ca in a given volume of the composite is assumed to be equal to the product of the projected area of the volume and the count-rate over that volume. The dimensions of the projected areas are shown on Figure 9, and are derived as follows:

Gap and overlap zones: width (50±4 nm) = average width of fibrils as inferred from BF images of longitudinal and cross-sections; lengths (40 nm and 27 nm respectively) are from known dimensions of these zones in collagen fibrils [39], [40], and confirmed by measurements here.

Mineral structures: The average width of the stack of mineral structures between fibrils is 28±10 nm wide. The length is arbitrarily terminated at the edge of the unit volume, although the mineral structures extend much farther parallel to the fibril.

From these dimensions we obtain the following approximate areas (in nm^2^): A(V) = 1810; A(G) = 2000; also, A(H) = 5160 (the area of the over/underlying MS within the entire unit volume) (figures have been rounded off).

We can now see that
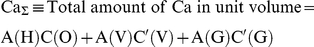


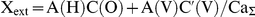
Using data shown in Table 3, we find that 81±4% of the mineral is external to the collagen fibrils. This result is consistent with the suggestion of Sasaki et al [25] and also with that of Bonar et al. [20] that at most 35% of the mineral in mature bovine bone could be within the fibrils. Models of bone structural behavior also show that a large portion of the mineral must be extrafibrillar to obtain the observed properties of bone as a material [23]. This large percentage of external mineral contradicts the gap zone mineral model [2], [7], [13].

**Table 3:** Count rates of Ca X-rays from EDXS measurements on selected areas of longitudinal section (Figure 5).

Sample Area	Sample 1	Sample 2	Sample 3	Units
C(O)	1.66	3.16	3.16	Counts/nm^2^
C(G)	4.55	5.2	5.2	Counts/nm^2^
C(V)	7.02	6.03	7.37	Counts/nm^2^
C′(G)	2.89	2.04	2.04	Counts/nm^2^
C′(V)	5.36	2.87	4.21	Counts/nm^2^
X(ext)	0.7595	0.8405	0.8543	%

Note - Sample areas (C(O), C(G), etc.) are defined in the text.

### Comparison with microtomed sections

Comparison of images obtained by ion milling in this study with analogous published images of sections of ultramicrotomed fully mineralized cortical bone suggests that the latter method results in damage to the structure of bone that has led to some previous confusion about the ultrastructure of bone. During ultramicrotoming, a sharp blade is used to cut a 100 nm-thick slice from the sample. Intense stress develops at the cutting edge during this process. Our model for bone ultrastructure would predict that the bundles of relatively rigid mineral structures, having very different mechanical properties from the collagen fibrils, may break into fragments whereas the softer fibrils would maintain their initial form. This might render certain key features of the original structure unrecognizable.

**Figure 10:**
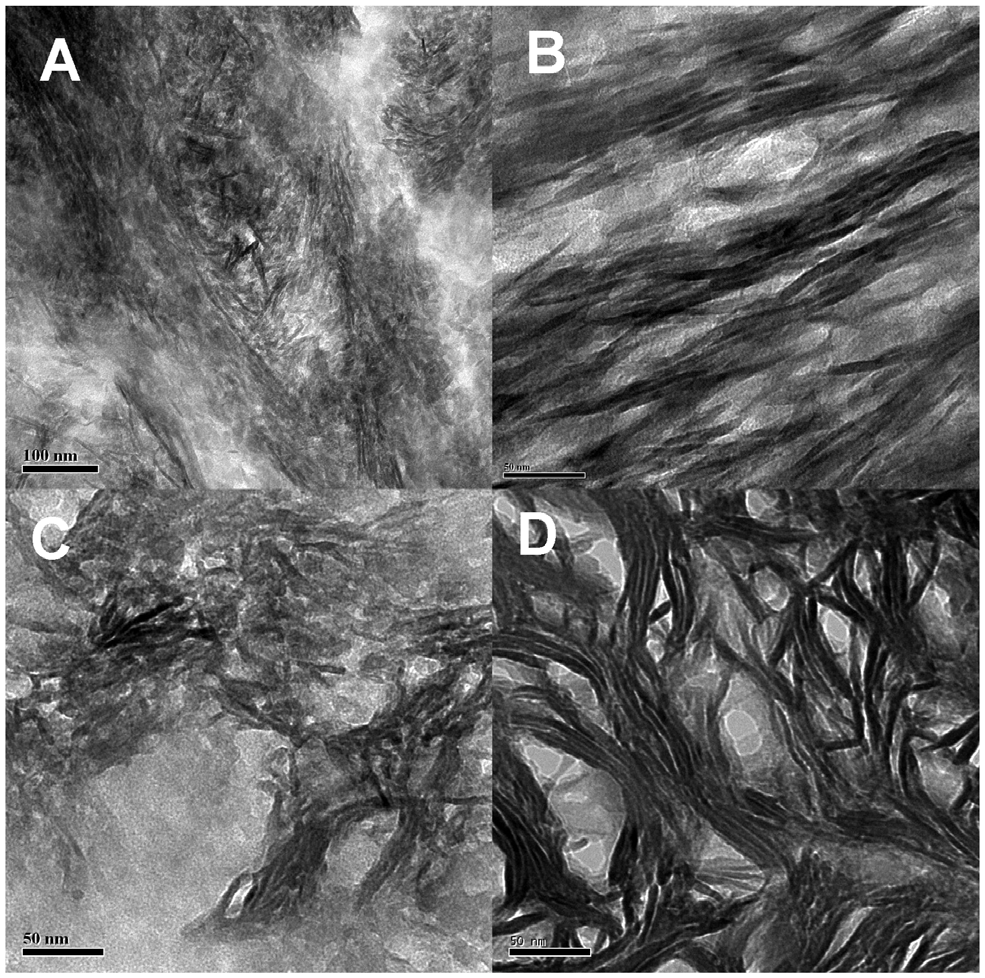
Comparison of sections produced by ultramicrotoming and ion milling. a) longitudinal section of bovine femur, scale = 100 nm; b) ion-milled section of same sample in same orientation, scale = 50 nm; c) ultramicrotomed cross-section of same bone, scale = 50 nm; d) ion-milled section of sample in same orientation, scale = 50 nm.

To test this hypothesis, samples of the cortex of a bovine femur were ultramicrotomed by an experienced technician in the electron microscopy laboratory of the Faculty of Health Sciences, McMaster University. Figure 10a presents a typical TEM image of an ultramicrotomed longitudinal section. Fragments of mineral structures can be seen, locally co-aligned, but we see no long-range alignment of these fragments. The average length of these fragments of mineral structures is 37±12 nm (n = 10). An ion milled section of the same bone in the same orientation (Figure 10b) clearly shows the collagen banding and mineral structures oriented perpendicular to these bands. The mineral structures are much longer than those in the ultramicrotomed section, suggesting that, as hypothesized, structures in the former section have been shattered during the cutting of the section.

Figure 10c shows a cross-section (perpendicular to the femoral axis) of the same bone prepared by ultramicrotoming. The open structure seen in ion milled sections is only vaguely perceptible here. The section resembles Lees et al.'s Figure 1b [21], a study in which samples were prepared by ultramicrotoming. The average length of the fragments of mineral structures present in Figure 10c is 26±12 nm (n = 10). An ion milled section of the same bone (Figure 10d) shows structures comparable to those seen in sections of human bone. The average length of mineral structures in this section is 54±18 nm (n = 10), double that seen in the ultramicrotomed section.

The tendency of mineral structures to be shattered by the action of the ultramicrotome knife presumably reflects their relative stiffness compared to the collagen fibrils. This would be especially true if relatively small amounts of hydroxyapatite were present inside the fibrils as we have suggested here. In earlier studies of bone, damage to internal structures during ultramicrotoming may have contributed to erroneous inferences about the location and orientation of the mineral with respect to in a fibrils.

### Nature of the mineral crystals

Several other studies have attempted to determine the size and shape of the crystals of HA in bone. One approach has been to remove collagen from bone particles by attack with an oxidizing agent such as hydrazine or NaClO [9], [41]–[43]. Neither of these reagents would be capable of dissolving the mineral phase. The resulting residue consists of flake-like particles <1 nm thick and 30–40 nm in diameter [9], [42]. SAD analysis shows that these are single crystals of apatite. Clearly, they must also be present in the samples which we studied. We hypothesize that these smaller, flake-like crystals are enclosed within the mineral structures in some way. In some longitudinal sections, in the zones between the lanes of mineral structures, we observe irregular boundaries spaced on a scale of 10's of nm which may demarcate individual crystals (Figure 2, arrows). Also, Moiré patterns present in these regions may represent overlapping single crystals whose lattices are not precisely co-aligned. Furthermore, the dark-field images (Figure 4b) showing discrete sub-regions with different lattice orientation may reflect the presence of these flakes within the mineral structures. This raises the question how these flakes are bound together to form the mineral structures.

The ultrastructure of bone which we have described here resembles in many ways that first proposed by Lees et al. [21] and more recently suggested for dentine by Jantou-Morris et al. [28], [29]. In this scheme, HA occurs as rigid struts oriented parallel to the collagen fibrils but external to them. This “design” would have a significant impact on calculations of the strength of the entire assemblage, especially in resisting axial loading (e.g. [4]) and should be incorporated into attempts to calculate the dynamic properties of bone (e.g. [18], [44]). The structure proposed here would appear to make better use of the strength of the HA than a design in which HA crystallites were enclosed within the fibrils.

Sections of other vertebrates shown above share the features which we have detailed using human femoral bone, although none have been examined here in sufficiently detail to define their similarities and differences. Nevertheless, it appears that the pattern of mineral structures external to and forming a carapace around collagen fibrils is a persistent and conserved motif in all vertebrates.
